# The broad range of self-management strategies that people with rheumatic and musculoskeletal conditions apply: an online survey using a citizen science approach

**DOI:** 10.1007/s00296-025-05842-2

**Published:** 2025-05-06

**Authors:** E. te Braake, R. Schriemer, C. Grünloh, S. Ahoud, T. Asselberghs, V. Bodelier, D. Hansen, C. Ophuis, R. Wolkorte

**Affiliations:** 1 Roessingh Research and Development, Enschede, The Netherlands; 2 Biomedical Signals and System Group, Faculty of Electrical Engineering, Mathematics, and Computer Science, University of Twente, Drienerlolaan 5, 7522 NB Enschede, The Netherlands; 3 Sint Maartenskliniek, Nijmegen, The Netherlands; 4 Radboud Universiteit, Nijmegen, The Netherlands; 5 On behalf of all patient patners within the REIS project, Enschede, Nijmegen, The Netherlands; 6 Health Technology and Services Research, Faculty of Behavioural, Management, and Social Sciences, University of Twente, Enschede, The Netherlands

**Keywords:** Self-management, Self-management strategies, Rheumatic and musculoskeletal disease, Citizen science, Surveys and questionnaires

## Abstract

Rheumatic and musculoskeletal diseases (RMDs) cause several restrictions in daily living. Self-management is an important aspect of managing RMDs. However, little is known about the self-management strategies that are currently applied in daily life. This study aimed to identify the current self-management strategies that people with RMDs apply through a citizen science approach. An online survey was iteratively developed together with people with RMDs. The survey was distributed among people with all types of RMDs. Survey responses were collected within Qualtrics, and once anonymized, analysed using Atlas.ti. General self-management strategies and motivations to start performing a strategy were deductively coded by two reviewers, after consultations with patient partners. 250 complete surveys were collected. 91.2% of the respondents were female. 1305 self-management strategies were mentioned, and 669 elaborations were given. Most participants applied self-management strategies within the ‘physical activity’ category in their daily lives (e.g., walking, biking). Motivations to start performing a certain self-management strategy mostly originated from the bodily functioning dimension (e.g., reducing pain). 1275 facilitators to start a self-management strategy were mentioned, which were mostly related to the ‘support’ category. Barriers (N = 480) were most frequent in the ‘condition-related’ category. Self-management is an important aspect of managing a person’s condition in daily life. People choose one or several strategies based on the challenge they are facing, depending on their feasibility and preferences in line with their personal context. The comprehensive overview of strategies informs both patients and healthcare professionals to support a personalized self-management journey.

**Supplementary Information:**

The online version contains supplementary material available at 10.1007/s00296-025-05842-2.

## Introduction

Rheumatic and musculoskeletal diseases (RMDs) entail a wide range of degenerative, inflammatory, and auto-immune conditions, that commonly affect the joints [1]. People with RMDs experience a high disease burden [2–4], and face multiple everyday challenges that are complex and interrelated [5]. Complaints such as fatigue, anxiety, and chronic pain are common among people with RMDs thereby affecting daily life activities and impacting their quality of life [6–11]. Often, medication is offered to deal with and manage symptoms. Several recommendations are made to complement pharmacological treatment, such as a healthy lifestyle and patient education [12, 13]. Thereby, indicating the importance of the responsibility of the patient to become an active participant in their care and carry out these lifestyle recommendations and hereby engage in self-management.

This paper defines self-management as: ‘‘The ability of an individual to manage one’s symptoms, treatment, physical, social, and emotional consequences, and lifestyle changes. It includes means of empowerment, educating oneself, being autonomous, learning and adapting to new behaviours, acceptance, and adapting to a new balance in life” [14]. People with chronic conditions differ in their support needs for self-management [15]. A previous scoping review showed that people with rheumatoid arthritis expressed the need for more informational, social, practical, and emotional support [16].

Self-management interventions in RMDs exert their positive effects (e.g., improvements in physical functioning and self-efficacy) [17–19]. However, even though self-management is considered an important aspect of managing one’s disease, little is known about which self-management strategies people with RMDs apply. As has been shown in research investigating self-management or self-care in Parkinson's, the activities that are part of the everyday life of a person with a chronic condition might not fit into a medicalized frame of self-care [20]. In addition, as patients are the health experts on all aspects of their lives, it might be very useful to look outside the clinical setting and look into the self-management practices of people living with the disease every day.

This study aimed to investigate which self-management strategies people with RMDs apply in their everyday lives utilizing a cross-sectional survey. Both positive and negative experiences with the strategy were collected. With such a survey, the self-management activities in and outside the clinical setting can be captured, thereby potentially striving towards creating a complete overview of day-to-day self-management from a patient perspective. Such insights may inform and inspire other patients in their journey to self-management, guide clinicians in increasing their knowledge which may contribute to providing patients with the right self-management support, and encourage researchers to form new hypotheses for developing evidence-based interventions.

## Methods

A citizen science approach was followed throughout the study to ensure that the outcomes are relevant for the target group, that the design of the study is feasible, and that we ask the right questions to yield high-quality results. Citizen science encompasses a range of participatory models for involving patient partners as collaborators in scientific research [21]. In this study, people with RMDs were considered partners in the project. The patient partners were people with at least one rheumatic condition, who were not employed by the research units. No specific training or skills were required to participate as a patient partner, other than being able to join the online meetings. Some had been involved in previous projects and/or had formal or informal training on different aspects of research. Patient partners were provided with a gift voucher after each meeting they attended to show the researchers’ appreciation. Throughout the project, 16 patient partners were involved. Each meeting was attended by between 4–8 patient partners and 2–5 researchers (/research assistants). Every step in the study was decided upon in co-creation between researchers and people living with RMDs: formulating research questions, setting up the study design, finding the most suitable ways for data collection and analysis, developing and testing the survey, interpreting the results, and communicating results. The data management plan was also formulated in co-creation. After a decision was made, the work was carried out by the researchers.

### Study design and survey development

**Fig. 1:**
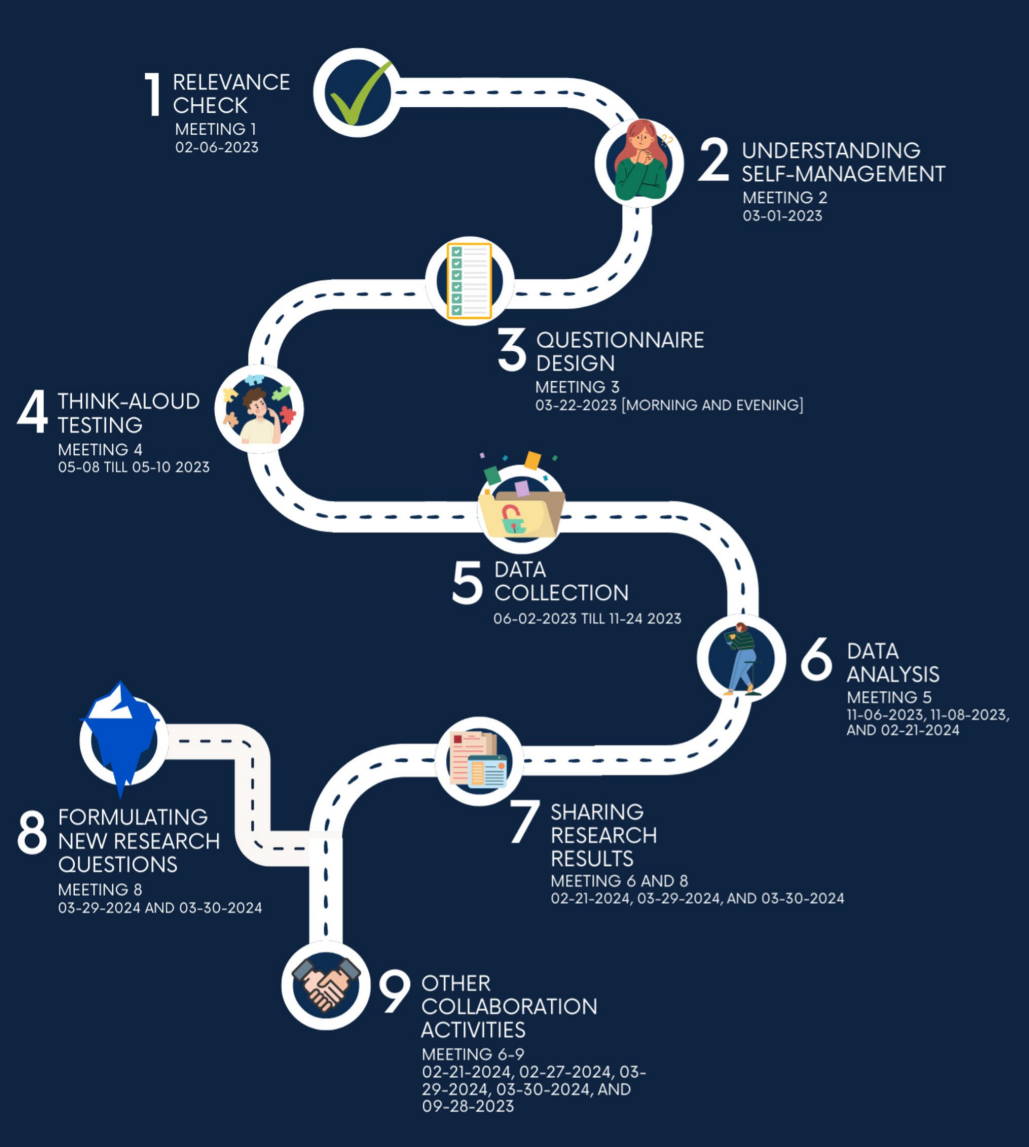
Roadmap of activities within the REIS project

The cross-sectional study consisted of an online survey. This study followed the items of [22] for reporting survey studies. As existing self-management surveys in literature (e.g., [23–25]) did not address this specific topic, we iteratively developed our own survey. Figure 1 shows the process of this project involving patient partners in every research stage. Before starting with the design of the study, we reached out through our existing network to people living with RMDs to join us in an initial meeting. In the first meeting with 7 patient partners and 4 researchers, we collaboratively explored the relevance of doing such research for identifying self-management strategies (Phase 1: Relevance check). Thereafter, the second meeting with a total of 6 partners and 3 researchers took place to better understand what self-management means for people living with a rheumatic condition (Phase 2: Understanding self-management). The survey itself was designed together with patient partners (N = 8), and researchers (N = 3) during the third meeting (Phase 3: Questionnaire design). Based on this co-creation, the first version of the online survey was developed using Qualtrics (Qualtrics, Provo, UT). This version was used as input for the fourth meeting in which one-on-one online think-aloud sessions with 3 patient partners were carried out (Phase 4. Think-aloud testing). Unclear sentences, complicated wording, and errors were identified during these sessions and improvements were made to develop the final version of the survey. After testing, the survey was rated as highly relevant and feasible by co-researchers. In total, 16 patient partners collaborated with us with varying frequencies.

### Survey

The survey consisted of 3 domains. First, questions regarding self-management strategies that people considered were asked (domain 1, 3–5 questions). Optionally, people could expand to describe their experiences, motivations to start, barriers, and facilitators (domain 2, 16 questions in case of one elaboration). Finally, demographic data was collected (domain 3, 14 questions). Completing the survey took 20–30 min on average. The final version of the online survey can be found in Supplementary Materials 1.

### Survey distribution

The survey was launched on the 2nd of July 2023 and closed on the 24 th of November 2023 (Phase 5. Data collection). A convenience sample of people with RMDs was targeted. The survey was shared through multiple channels including online outlets (e.g., social media channels, email), hospitals, patient organizations, newspapers, and universities. For a complete overview, see Supplementary Materials 1.

### Data preparation

Survey responses collected in Qualtrics were exported to a secured Excel file where data was anonymized and incomplete survey responses were deleted, as stated in the informed consent form. Survey responses were considered complete when participants filled in all obligated demographics questions at the end of the survey. This was also clearly communicated to participants in the information given at the start of the survey, providing an opt-out option during survey participation. All survey responses were anonymized by one author and 2 student assistants (EtB, KvM, YS), any information that could be traceable to a particular person was removed and replaced by the topic of that information (e.g., [place], [name]). The anonymized dataset is available upon reasonable request from the researchers [26].

### Data analysis

To describe the participants' characteristics, the mean, or range was calculated. Initially, we aimed to deductively code the data by using the “Taxonomy of Everyday Self-management Strategies” (TEDDS) [27] for the different strategies that people shared (i.e. “What did they do?”), and to deductively code the motivations behind applying the self-management strategy using the positive health model [28] (i.e., “Why did they do it?”). This was discussed and jointly agreed upon during one of the meetings. Twenty survey responses were separately coded by two reviewers (YS, EtB) using these two models, already raising questions about the mutual exclusiveness of the categories within the TEDDS model while doing so. After this initial coding, four online one-on-one meetings were organized to discuss the codes with patient partners (Phase 6: Data analysis). The objective of this meeting was to verify whether the codes assigned to the data aligned with how the patient partners interpreted these codes and whether they would assign the same codes. During these meetings, it became clear that the positive health model was suitable to use, but the TEDDS model used for the general part of the survey was much harder to fit the data that was collected. There was too much overlap between different self-management categories as they were not mutually exclusive (e.g., healthy behaviour and disease-controlling strategies) and also did not match the everyday language and frame of reference of people with RMDs. Together, we concluded that by using this model, the goal of this survey would not be achieved; namely, creating an overview of which self-management strategies people currently apply which is easily interpretable not only for academics but also for healthcare professionals (HCPs) and people with RMD’s. Thus, after several discussions within the research team, it was concluded that an inductive approach to analyse the data was more appropriate. Given that the researchers had already familiarised themselves in-depth with the data, a set of codes with definitions was developed and discussed. With this new coding scheme, the two coders (YS, EtB) coded the first 30 survey responses independently. Thereafter, a discussion took place to compare the coded data. The Krippendorff’s c-Alpha-binary was calculated to be 0.819 indicating good inter-rater reliability. Discrepancies in coded data were solved through discussion to reach a consensus between the coders, and categories and definitions were revised to be precise and exclusive. This resulted in the categorisation shown in Table 1, which was also discussed with patient partners who agreed that these categories were suitable and clear. Thereafter, the remaining survey responses were divided between the two coders (YS and EtB) and were coded separately.

**Table 1:** Self-management categories used to analyse data following the question “What did they do?”

Category	Definition
*Energy distribution*	Strategies that are used to use energy more efficiently by adjusting, spreading, or controlling the activity level with the aim of being able to continue to carry out daily activities
*Physical activity*	Physical activities performed to maintain, and/or improve health and vitality in daily life
*Nutrition and supplements*	All dietary lifestyle adjustments and/or lifestyles including the use of vitamin or mineral supplements
*Healthcare professionals*	Care by accredited health care professionals aimed at improving your body functions and maintaining your health such as physical therapy or occupational therapy
*Mental health*	Mental/cognitive internal strategies or beliefs used with the aim of accepting the consequences of the illness and/or generating mental peace
*Participation*	(Social/leisure) activities to (continue to) participate and/or contribute to society
*Medication*	Condition-specific medication and/or medication for symptom management/prevention
*Assistive devices*	Objects or services that support [participation in] daily activities
*Alternative medicine*	Alternative or complementary treatments, therapies, means, and techniques that deviate from conventional medical care and/or are not recognized as a medical profession/therapy/means with the aim of relieving symptoms
*Information and patient education*	Searching for information about the disease, self-management, solutions, and problems in order to gain more knowledge. In addition, specifically education and learning about the disease and its consequences
*General lifestyle alterations*	All general strategies to make lifestyle adjustments that do not fit the other categories and were not further specified
Other	Other self-management strategies that do not fall under any of the above-mentioned categories

The motivation why people apply a certain self-management strategy was an optional part of the in-depth elaboration questions. The motivational aspect was then coded using the model of positive health [28] as planned from the beginning. This model characterizes health as ‘the ability to adapt and self-manage in the face of social, physical, and emotional challenges’ and entails six dimensions, which are outlined in Table 2.

**Table 2:** Dimensions of positive health [29] used to analyse data following the question “Why did they do it?”

Category	Definition (Authors’ translations from examples of Positive Health Dialogue Tool 2.0)	Examples from Positive Health Dialogue Tool 2.0
*Bodily functions*	The primary motivation is health. This dimension focuses on movement, sleep, and symptoms and pain	Feeling healthy, feeling fit, no physical complaints and/or pain, sleeping, eating, sexuality, physical condition, physical activity
*Mental well-being*	A person's state of mind. In addition, rest and relaxation are also central here	Being able to: remember things, concentrate, communicate, handle change, be cheerful, accept yourself, feel in control
*Meaningfulness*	About lust for life. Alongside this is the importance of faith and religion, or the search for it	Having a meaningful life, having a zest for life, pursuing ideals, feeling confident, accepting life, being grateful, lifelong learning
*Quality of life*	The focus is on a new perspective on life with the disease	Enjoyment, being happy, feeling good, feeling well-balanced, feeling safe, intimacy, housing circumstances, having enough money
*Participation*	Participation is about participating in society and being able to engage in social activities. But also getting support and asking for help from the social environment	Social contact, being taken seriously, doing fun things together, having support from others, sense of belonging, doing meaningful things, being interested in society
*Daily functioning*	Daily functioning has as the primary motivation to continue to function in daily/ordinary life	Taking care of yourself, knowing your limitations, knowledge of health, managing time, managing money, being able to work, being able to ask for help
*Other*	Motivations that could not be applied to one of the other dimensions	

The possible categories for barriers and facilitators were included in the survey so that participants could fill in their experience directly in the categories of ‘knowledge’, ‘time’, ‘condition-related’, ‘money/compensation’, ‘support’, or ‘others’. Responses were carefully analysed and where applicable, reassigned to the correct category. Responses in the ‘others’ category were revised to decide whether they could warrant a separate category.

### Other collaborations and citizen science activities

During meetings 6 and 8, results were shared (Phase 7. Sharing research results) and potential questions for future research based on the outcomes of the current study were discussed (Phase 8. Formulating new research questions). As part of this citizen science approach, opportunities for potential collaborations in the dissemination phase were actively sought (e.g., going to conferences, designing research posters, co-authoring, interviews with patient magazines, among others). Therefore, during the different phases of this research, several meetings took place to organize and discuss these opportunities (Transcending phase: Phase 9. Other collaboration activities).

## Results

250 complete survey responses were collected. The results have no missing data as all replies were mandatory. A total of 1305 self-management strategies were reported by people with RMDs of which 669 (51,3%) were also elaborated on in-depth. Both strategies that participants experienced as positive (86%) and negative (14%) were mentioned. The amount of self-management strategies reported by a participant ranged from 1 to 10 strategies.

### Demographics

The majority of respondents were female (N = 228, 91.2%), with a mean age of 59.9. Most participants (60.1%) had high educational backgrounds with higher professional education being the most common. Osteoarthritis (N = 148) and rheumatoid arthritis (N = 94) were the most prevalent types of RMDs. Participants ranged in terms of year(s) living with the rheumatic condition(s) from less than a year to more than 20 years. Half of the participants also had comorbidities. An overview of all demographics collected within this study can be found in Supplementary Materials 2.

### Self-management strategies: What did they do?

Table 3 shows an overview of the self-management categories, including frequencies and examples. The results reveal that people who took part in the survey, deploy multiple strategies, covering a broad range. Most self-management strategies fit within the physical activity category. Strategies in the ‘other’ category, were not categorized as self-management strategies on itself, as they were mostly related to a process, such as patient empowerment, needed to perform such strategies. Either way, this process is still equally as important within self-management and therefore, worth mentioning.

**Table 3:** Frequencies and examples of self-management strategies in respective categories (total N = 1305)

Self-management strategy	N	Examples
*Physical activity*	260	Walking, **biking**, swimming, sports, staying active
*Assistive devices*	197	**E-bikes**, walking aids (e.g., frames, walkers), adapted kitchen knives, specific tools to open cans, regional transport services for people with disabilities
*Healthcare professionals*	146	**Physiotherapy sessions**, rehabilitation, occupational therapy, vitality coach
*Nutrition and supplements*	124	**Diet**, vitamin supplements, inflammation-reducing foods
*Mental health*	121	Acceptance following illness, **mindfulness**, meditation, speaking out about the rheumatic condition, and communicating personal boundaries timely
*Energy distribution*	109	**Taking more rest**, pacing, spreading tasks, planning, and taking breaks
*Participation*	108	**Employment or volunteering**, education, recreational time, hobbies, peer-support groups
*Alternative medicine*	84	Cannabidiol oil, **Homeopathic remedies**, attending the sauna, cold water exposure, self-hypnosis, hot bath, acupuncture
*Medication*	78	**Over-the-counter medication** (Analgesic medication, pain medication), prescribed RMD medication, and tapering off medication
*Information and patient education*	56	**Searching for information on the internet**, reading books about their rheumatic condition, following classes for a specific rheumatic topic, reading flyers
*General lifestyle alterations*	37	Losing weight, **changing/adapting lifestyle,** being healthy
*Other*	20	**Having a say in medical appointments**, being assertive with healthcare professionals and other organizations

The categories are ranked from most common to least common. Common examples are highlighted with bold text

### Motivation to perform strategy: Why did they do it?

In total, participants chose to elaborate in depth on N = 669 strategies. Part of this elaboration reflected the motivation on why they performed a certain strategy which was categorized using the positive health model (Table 4) [28]. Some strategies had more than one motivation. Most strategies (N = 421) fitted within the bodily functioning dimension. Not many motivations fitted in the ‘meaningfulness’ and ‘quality of life’ dimensions.

**Table 4:** Motivation for performing self-management strategies categorised in the Positive Health Dimensions, as part of the elaborations of N = 669 strategies

Category	N	Example quotes
*Bodily functioning*	421	‘Because the pain got worse, we switched to this [strategy]... [we] tried to see if it [the strategy] had an effect and **whether it reduced the pain’** (RSP018)
		‘**My muscles are always under tension**. I wanted to make my muscles stronger so that I would hopefully experience less and less pain and restlessness in my body’ (RSP071)
		‘I **would like to limit my dependency on medication**, so I try other ways to minimize the use of pharmacological means, I'm happy to do so’ (RSP036)
*Daily functioning*	135	‘Spread domestic chores in particular over a day or week. Rest periods in between. **No longer wanting to cram everything in one day**.... Energy runs out quickly’ (RSP1)
		‘Make a plan in advance, learn to respect and express my limitations... **Support for daily life**’(RSP39)
		‘I use a mobility scooter when we go out for a whole day with the family. I use earplugs when I am in a busy environment for a long time.... **Aids give me more freedom to do fun things and be less dependent**’ (RSP196)
*Mental well-being*	108	‘I found, and still find, it **difficult to accept that I am ill**. It feels weak. I hoped that yoga and mindfulness could help me with the process of accepting, to be able to live more in the present’ (RSP36)
		‘**To learn to accept** so that I would no longer overstep my boundaries and distribute my energy better’ (RSP183)
		‘To achieve **mental balance**’ (RSP66)
*Participation*	81	‘I would like **to mean something to the people around me**. If I am creative, this is also possible with limitations due to a chronic condition’ (RSP114)
		**Friends, family, movies, history lessons, and most importantly: work** and being busy. I forget the rheumatism’ (RSP228)
		‘**Contact with peers** and [to] gain more knowledge’ (RSP16)
*Quality of Life*	38	‘I **refuse to give up my freedom** if there is an opportunity to do so’ (RSP6)
		‘[To] Extend and i**mprove my quality of life**” (RSP48)
		‘Awareness of what is possible... because I like life,** I want to make something of it**’ (RSP228)
*Meaningfulness*	15	‘Reflecting on what is permanent in me, [on] **what my essence is**.’ (RSP114)
		‘I **find my life too passive**’ (RSP257)
		‘My job at the time had stopped at some point and I especially did not want to exclude myself from society and **dedicate my time and energy to advocacy for other people with RMDs**’ (RSP236)

The categories are ranked from most common to least common. Important parts of the quotes are highlighted with bold text

Some motivations were mentioned that were unrelated to any of the dimensions in the model and/or no motivation was provided in the responses. For example*, ‘Look at previous question’* (RSP202), or *‘Because many doctors and nurses just express unsubstantiated opinions*’ (RSP163). These ‘other’ motivations were so diverse that we could not identify a pattern or theme that would warrant an extra category.

### Facilitators and barriers

In the survey, we already provided categories for facilitators and barriers as multiple-choice options. During the analysis, it appeared that many experiences mentioned in the ‘other’ category were related to ‘emotional/mental processes’. Therefore, this was added as a separate category.

In total, 1275 facilitators were reported regarding 669 strategies (Table 5). Most facilitators were related to the ‘support’ category. Facilitators in the ‘other’ category were diverse and could not warrant a separate category. Some ‘other’ facilitators mentioned were, for example, about having positive experiences with a strategy and having no other choice than doing the strategy, among others. Furthermore, participants reported 480 barriers to starting a strategy (Table 5). Most barriers were related to the category ‘condition related’. Barriers mentioned in the ‘other’ category included, for example, bad experiences with a strategy, and having difficulties with persevering in a certain strategy.

**Table 5:** Experienced facilitators and barriers when performing self-management strategies

Category	Facilitators (N = 1275)		Barriers (N = 480)	
	*N*	Example quotes	*N*	Example quotes
*Knowledge*	223	**‘Reading other people's experiences and talking** to family members who also have rheumatism’ (RSP48)	28	‘**Not much was known** among other patients with RMDs about which self-medication worked best’ (RSP67)
		‘A **lot of knowledge gained** because of the occupational therapist’ (RSP101)		‘The information about this is **not clear**’ (RSP181)
*Time*	239	‘I am retired, so [I have] **enough time**’ (RSP9)	61	‘Applying for resources** takes a lot of time/energy**’ (RSP61)
		‘**I gave myself more tim**e because I didn't do things or did things differently that suited my situation better’ (RSP25)		‘**It takes a lot of time** to figure everything out’ (RSP183)
*Condition related*	206	‘Mindfulness gives direction and rest, [it] is **a decrease of disease activit**y’ (RSP12)	118	‘Physical activity is difficult at times because of** too much pain**’ (RPS129)
		‘Rheumatic **condition was stable**’ (RSP22)		‘Osteoarthritis **complaints are severe most of the time** and limit my actions’ (RSP103)
*Money/Compensation*	252	‘[The strategy] Was **fully reimbursed by insurance**’ (RSP2)	84	‘It [the strategy] **is expensive**, so you have to set priorities’ (RSP4)
		‘I had **enough money** to buy an e-bike’ (RSP52)		‘**Little reimbursement** from health insurer’ (RSP32)
*Support*	266	‘The substitute General Practitioner **took my complaints seriously**’ (RSP197)	89	‘It [the disease] **is often not understood**. I have lost quite a few “friends” because of this because they think I am acting out or not showing interest’ (RSP127)
		‘[My] environment **is supportive**’ (RSP252)		‘It is** difficult to find someone who can help** with this [the strategy] (RSP183)
*Emotional/Mental processes*	26	‘I wanted to **get my life back on track**’ (RSP129)	56	A bit of **acceptance that some things are no longer possible** or need to be done differently’ (RSP25)
		‘**Trusting your own feelings**, I do what feels right for myself’ (RSP56)		‘Emotion!** Emotion sometimes makes it difficult** to adjust your life’ (RSP41)
*Other*	66	‘**If your older when replacing prosthetics**, it you’re more likely to have complications’ (RSP5)	44	‘In **hot weather they are less comfortable** and give off because you sweat’ (RSP153)
		‘I slowly began to **experience the positive effects**’ (RSP50)		‘Getting started is not that difficult. **Persevering is what it’s all about**’ (RSP36)

The categories are ranked from most common to least common. Important parts of the quotes are highlighted with bold text

## Discussion

This paper identified a large number of diverse self-management strategies that people with RMDs apply in their daily lives. Many can be categorized as strategies related to ‘physical activity’ and ‘assistive device’ categories. Most strategies are initialized to improve bodily functioning. The paper provides information for patient organizations and healthcare professionals to educate and guide patients towards optimizing their personalized self-management activities.

### Self-management strategies

People with RMDs apply a variety of self-management strategies in their daily lives. Although it is known that clinical treatment is an important aspect of managing a chronic condition, many people apply additional non-pharmacological strategies [20, 30–32]. Specifically, participants in our study reported an average of 5 self-management strategies that they have tried and tested. In dialogue with our patient partners, it was emphasized that support in finding relevant self-management strategies is important, as they may not always be obvious or known to a large audience. Patient education and patient empowerment on this issue may help reduce disease burden and improve quality of life, especially for those with lower health literacy who are less likely to learn about specific strategies [33, 34]. Therefore, it is important that HCPs are aware of the efforts of people with RMDs, as they are often seen as their primary and trusted source of knowledge.

Although these self-management strategies occurred in diverse categories, most were in the physical activity category and mostly motivated by bodily or daily functioning. The prevalence of physical activity strategies might be explained by several reasons. First, the importance of an active lifestyle is quite well-known among the population and is often encouraged and recommended to them by HCPs [35–39]. Second, being active is relatively easy to incorporate in daily life (e.g., by doing home-based physical activity [40]. Third, this is one of the few strategies that is evidence-based and is known to alleviate physical symptoms such as pain and discomfort [41–43]. Notably, it might be the case that current systems to support self-management are designed to focus on supporting the physical aspect and that other aspects are underrepresented, as physical activity is the most consistent recommendation people receive from their HCP. Socio-psychological aspects are also very important when it comes to self-management and self-care [16], however, fewer motivations to start a self-management strategy were related to meaningfulness, participation, and quality of life. When discussing these results with our patient partners, they were surprised at how few motivations were recorded in the quality-of-life dimension. While it may be the case that people living with RMDs are less aware of certain strategies, our patient partners also mentioned that quality of life might be a dimension of positive health that is likely to be addressed indirectly. For example, one can easily imagine that assistive devices directly reduce symptoms and thus in the long run increase quality of life. Therefore, in future research, it is important to distinguish between immediate factors that are addressed and domains that are improved long-term as a result. Additionally, it is recommended to understand whether there is a lack of feasible interventions for certain goals, or whether some are actually more urgent than others.

People reported more facilitators that helped them to initialize a strategy, than barriers. Possibly, the presence of barriers would have prevented people from even considering a strategy and therefore not reporting it in our survey. Although some barriers were deemed condition-related, which are hard to target (i.e., fatigue-prohibited exercise), others have the potential for change. For example, it was mentioned several times that financial issues (i.e., no reimbursement from the insurance for physiotherapy, or to purchase assistive devices) served as a barrier. Health insurance companies and society, in general, should reflect on the barriers mentioned in this study and consider reimbursements if this would increase quality of life or self-sufficiency through self-management. Moreover, the barriers mentioned in this study could also clarify to HCPs why people are more or less inclined to practice self-management. HCPs should be aware that even when patients seem motivated to perform self-management strategies in their daily lives, barriers may present that restrict them from doing so (e.g., a lack of social support network). At the same time, knowledge was a strong facilitating factor, stressing the importance of educating people with RMDs about possible self-management options. Therefore, we invite HCPs to be aware of these barriers and facilitators when discussing and recommending self-management activities to their patients.

### The methodological approach used for data analysis

A previously developed self-management model by [27] did not suit the rich data in the current study. Therefore, after careful consideration and multiple discussions with patient partners, we iteratively formulated our own self-management categories derived from the data in our study. Using an inductive approach and creating our own categories was the most suitable approach to reflect the wealth of data and fit the needs of people within our project and hopefully, the wider population of people with RMDs. Furthermore, the rising prevalence of comorbidities indicates that self-management should preferably be symptom- or problem-oriented rather than condition-oriented. Therefore, it is interesting to investigate whether the self-management categories that we developed, also apply to other patient groups exceeding the RMD population.

### Creating conditions for citizen science

Throughout the different stages of this study, people with RMDs were involved as patient partners [44, 45]. Some steps were designed together, and on other occasions, proposed processes and next steps were changed after a meeting took place with patient partners. Although the inclusion of the knowledge and expertise of patient partners added value to the project, it is important to note that it takes extra time and effort and should not be taken for granted. Therefore, we will share our joint lessons learned during this project. We experienced that both patient partners and researchers valued the close collaboration. According to patient partners, researchers listened to everyone and took input and feedback seriously. Decisions about this project were made after consultation with patient partners, which were always organized to be highly accessible with no training or preparation that was demanded in advance. Several actions facilitated relevant participation in all phases of the research. First, researchers provided clear information to patient partners before every meeting and gave them sufficient time in advance to read it. Second, researchers provided summaries of meetings and decisions so that people could check, give feedback, and/or add missing pieces, which simultaneously lowered the threshold for the people who weren’t able to participate in the meeting to participate the next time. Third, researchers established a sufficient community size (e.g., between 10 and 20 patient partners). This lowered the pressure and burden of participating in each round, while simultaneously enabling the group to complement each other and to represent multiple viewpoints [46]. The group size also increased a sense of belonging as patient partners felt supported by one another. Fourth, the researchers actively looked for opportunities for patient partners to increase their involvement and show appreciation for their involvement. This was done by inviting patient partners to participate in congresses, co-authoring publications, and offering gift vouchers as a way of compensation and appreciation for the expertise provided. Fifth, the trust and sense of common interest established by the steps above facilitated the sense of co-ownership of the project. To conclude, this participatory approach based on citizen science has had a huge positive impact on the current project.

### Limitations

This study provides an overview of self-management strategies that people with RMDs apply. However, even though many people recorded their strategies, and the level of personal success they have had with these, this does not warrant any claims about their effectiveness on a group level. Thus, we cannot make recommendations to people with RMDs to perform particular self-management strategies. Furthermore, this study does not provide a complete insight into the needs of people with RMDs towards self-management. We only identified which strategies were carried out. Therefore, future research should identify whether additional self-management strategies are warranted and focus on investigating the effectiveness of these strategies mentioned in this study, to generate an evidence base to make specific recommendations.

In spite of the numerous online and offline distribution methods for recruiting participants, the survey itself was only available online. Although efforts were made to create an accessible survey, people with low digital skills were likely unable to participate in the survey. However, this study served as a starting point for identifying the current self-management strategies of people with RMDs, and we collected a rich dataset.

Finally, despite efforts to reach a large audience, the vast majority of participants have been living with their RMDs for several years, were female, and higher educated. This may jeopardize the generalizability of the results and one should be aware of this limitation when interpreting the results. The female dominance may be due to the online nature of this study, as previous research concluded that response rates for only surveys are higher among the female population [47]. As it is known that there are gender differences in coping and self-management strategies [48, 49], future research should investigate strategies of the male population by e.g., focusing on offline data collection methods.

## Conclusion

This study revealed that people with RMDs apply multiple and diverse self-management strategies in their daily lives. Most strategies were related to physical activity and the motivation to start a strategy is often to address a physical issue. This study showed that people with RMDs do much more for their health than solely following the clinical path and being passive recipients of medical care. As managing a chronic condition affects all aspects of one’s condition, management during everyday activities is needed. People with RMDs in this study are intrinsically motivated to improve their situation and take responsibility for their condition, by applying different self-management activities, and through trial and error, experiencing what works best for them to become active participants in their care. These findings are not only important for people with RMDs but also serve as an important foundation for clinicians to enhance their knowledge about self-management support.

## Supplementary Information

Below is the link to the electronic supplementary material.

Supplementary file1 (DOCX 112 KB)

Supplementary file2 (DOCX 28 KB)

## Data Availability

The authors confirm that the data supporting the findings of this study are available upon reasonable request from the researchers within the DANS Data Station Life Sciences repository, 10.17026/LS/KBBESE.
