# Supplementary material for: The broad range of self-management strategies that people with rheumatic and musculoskeletal conditions apply: an online survey using a citizen science approach
Source: Rheumatol Int. 2025 May 6;45(5):135. doi: 10.1007/s00296-025-05842-2 (PMC12055631; doi:10.1007/s00296-025-05842-2)
Supplement: Supplementary file 1 — Supplementary file1 (DOCX 112 KB) [file 296_2025_5842_MOESM1_ESM.docx]

# **Supplementary Material 1- Full survey [English] and dissemination channels**

**Article title**: The broad range of self-management strategies that people with rheumatic and musculoskeletal conditions apply: An online survey using a citizen science approach

**Journal name:** Rheumatology International

**Author names:** E. te Braake¹’², R. Schriemer³’^4^, C. Grünloh¹’², S. Ahoud^5^, T. Asselberghs^5^, V. Bodelier^5^, D. Hansen^5^, C. Ophuis^5^, R. Wolkorte^6^

**Affiliations:**

¹ University of Twente, Biomedical Signals and System group, Faculty of Electrical Engineering, Mathematics, and Computer Science, Enschede, the Netherlands

² Roessingh Research and Development, Enschede, the Netherlands

³ Sint Maartenskliniek, Nijmegen, the Netherlands

^4^ Radboud Universiteit, Nijmegen, the Netherlands

^5^ on behalf of all patient partners,

^6^ University of Twente, Health Technology and Services Research, Faculty of Behavioural, Management, and Social Sciences, Enschede, the Netherlands,

**Corresponding author:** Eline te Braake, [e.tebraake@utwente.nl](mailto:e.tebraake@utwente.nl)

REIS questionnaire


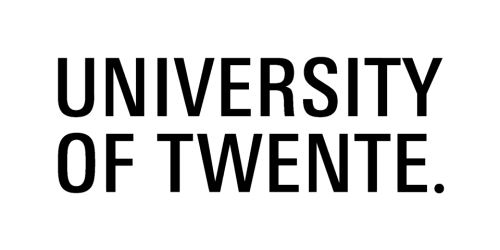

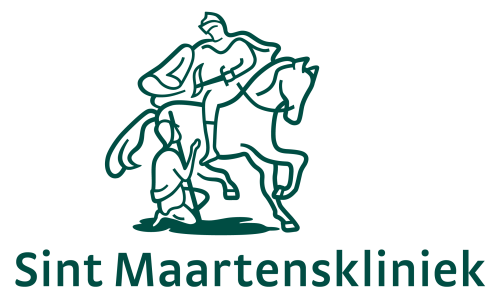

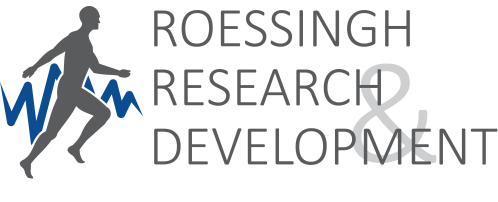


Dear participant,

You have a form of rheumatism, are over 16 years old, and are interested in participating in research into self-management in rheumatism. For self-management, people use different strategies (think of actions, skills, and use of tools). These are aimed at managing symptoms and consequences of the condition for your well-being and your active participation. Self-management is not just about 'doing it yourself'. It is also about 'self-determination'.

Many people go on self-examination to deal with the consequences of rheumatism. That probably applies to you too. With the knowledge and experiences about what helped you and others with rheumatism – or not! - we help people in their search. Our research is not aimed at determining what works for patients in general. We do want to learn from people, what they do and what their experiences are.

**The questionnaire**

In the questionnaire we collect your experiences with certain activities, methods, tools, etc. We call these activities, methods, and tools strategies. The questionnaire consists of three parts. First, we ask you to describe some strategies you have tried (up to 10). We ask you for both the positive and negative strategies experienced. This way, we can create an overview of barriers and facilitators. Then, you indicate which of these strategies you want to elaborate upon. We end the questionnaire with some information about yourself. Completing the questionnaire will take about 20-30 minutes.

The questionnaire largely contains open questions. That means that you can type the answers into a text field yourself. This way you can better express what you want to share so we get a better understanding of your considerations and experiences. Your written answers matter!

**Tips**

We also know that typing can be difficult when you have sore hands. We recommend that you do the research at a time when you have enough time and energy and are in a quiet environment. You can then think quietly and type comfortably. We think that typing on a PC, laptop or tablet is a bit easier than on a phone. You can stop for a while during filling in the questionnaire. Your answers are automatically saved. You can then return to the questionnaire later. Of course, you are not obliged to participate in the study, and you can stop at any time. Only the questionnaires that are completed until the end are included in the analysis.

**Personal data**

This study has been approved by the Ethical Review Committee of the University of Twente. This means that this research is carried out scientifically carefully and in accordance with your rights as a participant.

The data is stored at the University of Twente. Your data is well protected. We do not ask you for any traceable information. So: no names, no housing details, or data of others. We think it is important to work openly. That is why we place the anonymized data in online storage. This means that other researchers can contact us to view this data and use it for scientific research or education. We only give access if the scientific researchers have a suitable question.

The results of the research will be published in a scientific journal and of course also shared with important organizations that work for and with people with rheumatism. If you want to be kept informed of the research results, you can share your email address with us. This is stored separately from your answers.

If you have any questions about the study, you can contact [name of researcher] at [email researcher]

INFORMED CONSENT

Q1

- Yes, I hereby declare that I have read the information above, and I agree to participate in this study. (1)

Q2

Are you 16 years or older and do you have a form of rheumatism?

Not sure? Then take a look at the site of Rheumatism Netherlands

- Yes, I am 16 years or older and have a form of rheumatism (1)
- No, I am not 16 years or older and/or do not have any form of rheumatism (2)

Display This Question:

Are you 16 years or older and do you have a form of rheumatism?  Not sure? Then take a look at the sit... = No, I am not 16 years or older and/or do not have a form of rheumatism

Q3

Unfortunately, this questionnaire is not intended for you. This questionnaire is only intended for people who are 16 years or older and have a form of rheumatism.

Skip To: End of Survey If Unfortunately, this questionnaire is not intended for you. This questionnaire is intended for people only... Displayed

Display This Question:

Are you 16 years or older and do you have a form of rheumatism?  Not sure? Then look at the sit... = Yes, I am 16 years or older and have a form of rheumatism

SELF-MANAGEMENT GENERAL

Q4

This part of the questionnaire focuses on the self-management of your rheumatism. In the following questions you can name which strategies you want to share.

*Self-management strategies are actions that you perform aimed at dealing with rheumatism, complaints, and emotions yourself. Think, for example, of what you have done yourself to move forward, how you deal with the rheumatism, and what methods and solutions you have applied.*

After this part, you will be invited to provide more information about one or more of the strategies mentioned.

Q6

What self-management strategies do you use, or have you used in the past? Describe the strategy in no more than two or three words.

*These can be strategies that you have had good or bad experiences with. Think, for example, of aids, information, mental health, exercise, and so on.*

- (1) __________________________________________________
- (2) __________________________________________________
- (3) __________________________________________________
- (4) __________________________________________________
- (5) __________________________________________________

Carry Forward Entered Choices - Entered Text from "What self-management strategies do you use, or have you used in the past? Describe the strategy in no more than two or three words. These can be strategies that you have had good or bad experiences with. Think, for example, of aids, information, mental health, exercise and so on. "

Q7

Was this strategy mainly a good or bad experience?

|  | Good (1) | Bad (2) |
| --- | --- | --- |
| (x1) |  |  |
| (x2) |  |  |
| (x3) |  |  |
| (x4) |  |  |
| (x5) |  |  |

Q8

Do you have any other self-management strategies that you haven't mentioned yet?

- Yes, I want to name more strategies (1)
- No, I have mentioned everything (2)

Skip To: End of Block If Do you have any more self-management strategies that you haven't mentioned yet?  = No, I have mentioned everything

Display This Question:

Do you have any more self-management strategies that you haven't mentioned yet?  = Yes, I want to name more strategies

|  |
| --- |

Q9

What self-management strategies do you use, or have you used in the past? Describe the strategy in no more than two or three words.

*These can be strategies that you have had good or bad experiences with. Think, for example, of aids, information, mental health, exercise, and so on.*

- (1) __________________________________________________
- (2) __________________________________________________
- (3) __________________________________________________
- (4) __________________________________________________
- (5) __________________________________________________

Carry Forward Entered Choices - Entered Text from "What self-management strategies do you use, or have you used in the past? Describe the strategy in no more than two or three words. These can be strategies that you have had good or bad experiences with. Think, for example, of aids, information, mental health, exercise, and so on. "

|  |
| --- |

Q10 Was this strategy mainly a good or bad experience?

|  | Good (1) | Bad (2) |
| --- | --- | --- |
| (x1) |  |  |
| (x2) |  |  |
| (x3) |  |  |
| (x4) |  |  |
| (x5) |  |  |

Carry Forward Entered Choices - Entered Text from "What self-management strategies do you use, or have you used in the past? Describe the strategy in no more than two or three words. These can be strategies that you have had good or bad experiences with. Think, for example, of aids, information, mental health, exercise and so on. "

|  |
| --- |

SELF-MANAGEMENT ELABORATION

Q11

Which of these strategies do you want to elaborate upon?

*Choose which one and how much you want to work out. Each elaboration takes about 5 minutes.*

- (1)
- (2)
- (3)
- (4)
- (5)

Display This Question:

Do you have any more self-management strategies that you haven't mentioned yet?  = Yes, I want to name more strategies

Carry Forward Entered Choices - Entered Text from "What self-management strategies do you use, or have you used in the past? Describe the strategy in no more than two or three words. These can be strategies that you have had good or bad experiences with. Think, for example, of aids, information, mental health, exercise and so on. "

|  |
| --- |

Q12

Which of these strategies do you want to elaborate upon?

*Choose which one and how much you want to work out. Each elaboration takes about 5 minutes.*

- (1)
- (2)
- (3)
- (4)
- (5)

Q13

You will now work out the chosen strategies.

*You don't have to answer all the questions if you don't want to.*

Q14

What exactly did you do for ${lm://Field/1}?

________________________________________________________________

________________________________________________________________

________________________________________________________________

________________________________________________________________

________________________________________________________________

Q15

What was the reason you used this strategy called ${lm://Field/1} ?

________________________________________________________________

________________________________________________________________

________________________________________________________________

________________________________________________________________

________________________________________________________________

Q16

When did you do ${lm://Field/1}?

*Consider, for example, how your personal situation and/or the rheumatism situation was: just diagnosed, children, retirement, travel plans.*

________________________________________________________________

________________________________________________________________

________________________________________________________________

________________________________________________________________

________________________________________________________________

Q17

What are your experiences with ${lm://Field/1}?

- Very negative (1)
- Mainly negative (2)
- Mostly positive (3)
- Very Positive (4)

Q18

What went well with the execution of ${lm://Field/1}?

________________________________________________________________

________________________________________________________________

________________________________________________________________

________________________________________________________________

________________________________________________________________

Q19

What made it possible to start with this strategy (${lm://Field/1})?

- Money/compensation *(for example*: "*I had enough money"),* because (1) __________________________________________________
- Time, *(for example, "I had plenty of time")* because: (2) __________________________________________________
- Support (*for example: "Someone could help me")* because: (3) __________________________________________________
- Knowledge, (*for example, "I have read information")* because: (4) __________________________________________________
- Condition-related, *(for example: "The rheumatism was/was not active")* because: (5) __________________________________________________
- Otherwise, because: (6) __________________________________________________

Q20

What went wrong with the execution of ${lm://Field/1}?

________________________________________________________________

________________________________________________________________

________________________________________________________________

________________________________________________________________

________________________________________________________________

Q21

What made it difficult to start with this strategy (${lm://Field/1})?

- Money/compensation, *(for example: "I had no money")* because: (1) __________________________________________________
- Time, (*for example: "I didn't have enough time off*") because: (2) __________________________________________________
- Support, *(for example, "No one could help me")* because: (3) __________________________________________________
- Knowledge, (*for example, "I didn't understand very well what to do*") because: (4) __________________________________________________
- Condition-related, *(e.g. "The rheumatism was/was not active")* because: (5) __________________________________________________
- Otherwise, because: (6) __________________________________________________

Q22

Do you want to say something about the strategy ${lm://Field/1}?

________________________________________________________________

________________________________________________________________

________________________________________________________________

________________________________________________________________

________________________________________________________________

Q23

What exactly did you do for ${lm://Field/1}?

________________________________________________________________

________________________________________________________________

________________________________________________________________

________________________________________________________________

________________________________________________________________

Q24

What was the reason you used this strategy called ${lm://Field/1} ?

________________________________________________________________

________________________________________________________________

________________________________________________________________

________________________________________________________________

________________________________________________________________

Q25

When did you do ${lm://Field/1}?

*Consider, for example, how your personal situation and/or the rheumatism situation was: just diagnosed, children, retirement, travel plans.*

________________________________________________________________

________________________________________________________________

________________________________________________________________

________________________________________________________________

________________________________________________________________

Q26

What are your experiences with ${lm://Field/1} ?

- Very negative (1)
- Mainly negative (2)
- Mostly positive (3)
- Very Positive (4)

Q27

What went well with the execution of ${lm://Field/1}?

________________________________________________________________

________________________________________________________________

________________________________________________________________

________________________________________________________________

________________________________________________________________

Q28

What made it possible to execute this strategy (${lm://Field/1})?

- Money/compensation *(For example*: *I had enough money),* because (1) __________________________________________________
- Time, *(for example, "I had plenty of time")* because: (2) __________________________________________________
- Support (*for example: "Someone could help me")* because: (3) __________________________________________________
- Knowledge, (*for example, "I have read information")* because: (4) __________________________________________________
- Condition-related, *(for example: "The rheumatism was/was not active")* because: (5) __________________________________________________
- Otherwise, because: (6) __________________________________________________

Q29

What went wrong with the execution of ${lm://Field/1} ?

________________________________________________________________

________________________________________________________________

________________________________________________________________

________________________________________________________________

________________________________________________________________

Q30

What made it difficult to start with this strategy (${lm://Field/1})?

- Money/compensation, *(for example: "I had no money")* because: (1) __________________________________________________
- Time, *(for example: "I didn't have time")* because: (2) __________________________________________________
- Support, *(for example, "No one could help me")* because: (3) __________________________________________________
- Knowledge, *(for example, "I didn't really understand what to do")* because: (4) __________________________________________________
- Condition-related, *(For example: "The rheumatism was/was not active")* because: (5) __________________________________________________
- Otherwise, because: (6) __________________________________________________

Q31

Do you want to say something about this strategy ${lm://Field/1}?

________________________________________________________________

________________________________________________________________

________________________________________________________________

________________________________________________________________

________________________________________________________________

DEMOGRAPHICS

Q32

We now ask for some information about your personal situation.

*We do this to get a good view of the participants and whether we have reached a good cross-section of people with rheumatism with our questionnaire.*

Q33

What is your year of birth?

▼ 1930 (1) ... 2007 (78)

Q34

What is your gender?

- Male (1)
- Woman (2)
- I'd rather not say (3)
- Other, namely: (4) __________________________________________________

Q35

Do you live alone?

- Yes (1)
- No (2)

Q36

What applies to you?

*There are several possible answers.*

- I work for a fee (4)
- I do volunteer work (5)
- I am a housewife/househusband/caregiver (6)
- I have taken (early) retirement (AOW, VUT, FPU) (7)
- I am unemployed/looking for work (registered with the UWV WERKbedrijf) (8)
- I am incapacitated for work (WAO, WAZ, WIA, Wajong, sickness benefit) (9)
- I have a social assistance benefit (10)
- I am in education/I am studying (11)
- Other, namely: (12) __________________________________________________

Q37

What is your highest degree?

- None (1)
- Primary school / primary education (2)
- MAVO/ MULO/ domestic science school/ VBO (3)
- HAVO/ HBS (4)
- VWO/ Atheneum/ Gymnasium (5)
- MBO/ MTS (6)
- HBO (7)
- WO or doctoral (8)
- Other, namely: (9) __________________________________________________

Q38

What is your country of birth?

________________________________________________________________

Q39

What is your mother's country of birth?

________________________________________________________________

Q40

What is your father's country of birth?

________________________________________________________________

Q41

How do you feel about your current financial situation?

*This information says something about opportunities to buy or deploy self-management strategies that are not free*

Rate this on a scale from 0 ("I'm very worried") to 10 ("I'm not worried at all").

|  | 0 (13) | 1 (16) | 2 (17) | 3 (18) | 4 (19) | 5 (20) | 6 (21) | 7 (22) | 8 (23) | 9 (24) | 10 (25) |
| --- | --- | --- | --- | --- | --- | --- | --- | --- | --- | --- | --- |
| (6) |  |  |  |  |  |  |  |  |  |  |  |

Q42

What form(s) of rheumatism do you have?

*There are several answers to this question.*

- Psoriatic Arthritis (1)
- Temporal arteritis (2)
- Osteoarthritis (3)
- Paget's Bone Disease (4)
- Chondrocalcinosis (5)
- Raynaud's Phenomenon (132)
- Fibromyalgia (133)
- Hypermobility (134)
- Juvenile rheumatism (JIA) (135)
- Gout (136)
- Lupus Erythematosus (137)
- MCTD (138)
- Mono-arthritis (139)
- Myosotis (140)
- Osteoporosis (141)
- Palindrome rheumatism (142)
- Reactive Arthritis (143)
- Rheumatoid arthritis (144)
- Sarcoidosis (145)
- Scleroderma (146)
- Muscular rheumatism (147)
- Sjogren's syndrome (148)
- Ankylosing Spondylitis / Axial Spondyloarthritis (149)
- Forestier's disease (150)
- Other, namely: (151) __________________________________________________

Q43

When were you diagnosed with rheumatism?

- Less than a year ago (1)
- 1-2 years ago (2)
- 3-5 years ago (3)
- 6-10 years ago (4)
- 11-20 years ago (5)
- Over 20 years ago (6)
- I don't know anymore (7)

Q44

When did you get your first rheumatism complaints?

- Well before diagnosis (one year or more) (4)
- Around the diagnosis (5)
- I had no complaints (6)
- I don't know anymore (7)

Q45

Do you have any other chronic diseases?

- Yes (1)
- No (2)

Skip To: End of Block If Do you have any other chronic diseases?  = No

Q46

What other chronic disease(s) do you have?

________________________________________________________________

CLOSING

Q47 Are there any additional points about self-management that you would like to share with us?

________________________________________________________________

Q48

This is almost the end of the questionnaire. When you go to the next page, your data will be sent. After that, you will be redirected to another website. Do you want to stay informed about the research? Then leave your details there. If not, you can click away from that page. Thank you for participating!

REIS Dissemination channels

The survey was shared with the websites of the Dutch Arthritis Foundation (ReumaNederland), the University of Twente, The National Association of Rheumatic Care Netherlands, Dutch rheumatic patient magazine (Reuma Magazine), the Dutch online care newspaper (de Zorgkrant), The Dutch national association for people with lupus, antiphospholipid syndrome, scleroderma, and mixed connective tissue disease (NVLE: Dutch patient association for Sjögren's syndrome (NVSP), the Dutch association for psoriasis (Psoriaris Vereniging Nederland), the Dutch association for Juvenile Rheumatism Arthritis (Jeugdreuma Vereniging Nederland), the panel from Sint Maartenskliniek and Radboudumc for patients to structurally participate in scientific research (STAP: Key to active participation policy), among members of the citizen science community Citizenlab, through flyers in the waiting rooms of the Sint Maartenskliniek Nijmegen, on LinkedIn, and on personal social media accounts from the researchers.
