# Supplementary material for: The broad range of self-management strategies that people with rheumatic and musculoskeletal conditions apply: an online survey using a citizen science approach
Source: Rheumatol Int. 2025 May 6;45(5):135. doi: 10.1007/s00296-025-05842-2 (PMC12055631; doi:10.1007/s00296-025-05842-2)
Supplement: Supplementary file 2 — Supplementary file2 (DOCX 28 KB) [file 296_2025_5842_MOESM2_ESM.docx]

## **Supplementary Materials 2. Demographics**

**Article title**: The broad range of self-management strategies that people with rheumatic and musculoskeletal conditions apply: An online survey using a citizen science approach

**Journal name:** Rheumatology International

**Author names:** E. te Braake¹’², R. Schriemer³’^4^, C. Grünloh¹’², S. Ahoud^5^, T. Asselberghs^5^, V. Bodelier^5^, D. Hansen^5^, C. Ophuis^5^, R. Wolkorte^6^

**Affiliations:**

¹ University of Twente, Biomedical Signals and System group, Faculty of Electrical Engineering, Mathematics, and Computer Science, Enschede, the Netherlands

² Roessingh Research and Development, Enschede, the Netherlands

³ Sint Maartenskliniek, Nijmegen, the Netherlands

^4^ Radboud Universiteit, Nijmegen, the Netherlands

^5^ on behalf of all patient partners,

^6^ University of Twente, Health Technology and Services Research, Faculty of Behavioural, Management, and Social Sciences, Enschede, the Netherlands,

**Corresponding author:** Eline te Braake, [e.tebraake@utwente.nl](mailto:e.tebraake@utwente.nl)

| Category | Subcategory | N |
| --- | --- | --- |
| *Gender* | Female | 228 (91.2 %) |
|  | Male | 22 (8.8 %) |
| *Age* | Mean | 60 |
|  | Range | 1933-1999 |
| *Educational level* | No formal education | 1 |
|  | Primary education | 3 |
|  | Pre-vocational secondary education | 29 |
|  | Senior general secondary education/pre-university education | 20 |
|  | Secondary vocational education | 49 |
|  | Higher professional education | 103 |
|  | University or doctorate | 39 |
|  | Other | 6 |
| *Type(s) of Rheumatic condition(s)* | Arthritis Psoriatic | 20 |
|  | Arteritis Temporalis | 2 |
|  | Arthrosis | 148 |
|  | Chondrocalcinosis | 1 |
|  | Raynaud’s phenomenon | 26 |
|  | Fibromyalgia | 40 |
|  | Hypermobility | 23 |
|  | Juvenile Idiopathic Arthritis | 7 |
|  | Gout | 7 |
|  | Reactive arthritis | 2 |
|  | Systematic Lupus Erythematosus | 7 |
|  | MCTD | 1 |
|  | Myositis | 1 |
|  | Osteoporosis | 12 |
|  | Palindromic rheumatism | 2 |
|  | Rheumatoid arthritis | 94 |
|  | Sarcoidosis | 1 |
|  | SCCH/SAPHO | 1 |
|  | Scleroderma/Systemic Sclerosis | 8 |
|  | Muscular rheumatism | 11 |
|  | Sjögren's syndrome | 24 |
|  | Bekhterev disease/ Axial spondylarthritis | 6 |
|  | Forestier's disease | 1 |
|  | Tietze's disease | 1 |
|  | Other | 2 |
| *Year(s) living with Rheumatic condition(s)* | Less than a year ago | 8 |
|  | 1-2 years ago | 13 |
|  | 3-5 years ago | 42 |
|  | 6-10 years ago | 59 |
|  | 11-20 years ago | 69 |
|  | More than 20 years ago | 56 |
|  | I don’t remember | 3 |
| *Comorbidities* | Yes | 125 |
|  | No | 125 |
